# CLEVER-1^+^ tumor-associated macrophages in gastric cancer immunotherapy resistance: integrating metabolic reprogramming and immunosuppression

**DOI:** 10.3389/fimmu.2026.1913444

**Published:** 2026-09-22

**Authors:** Hao Yuan, Nan Li, Huaijue Qiu, Lin Wang, Yachao Li

**Affiliations:** 1 Department of Gastrointestinal Surgery, Chengdu Seventh People ‘ s Hospital (Affiliated Cancer Hospital of Chengdu Medical College), Chengdu, China; 2 GI Medicine, Binhai Hospital Of Tianjin Medical University General Hospital, Tianjin, China; 3 School of Medicine, University of Electronic Science and Technology of China, Chengdu, China; 4 Department of Gastrointestinal Surgery, Sichuan Academy of Medical Sciences & Sichuan Provincial People’s Hospital, University of Electronic Science and Technology of China, Chengdu, China; 5 School of Medicine and Life Sciences, Chengdu University of Traditional Chinese Medicine, Chengdu, China

**Keywords:** CLEVER- 1, gastric cancer, metabolic reprogramming, PD-1 inhibitor resistance, tumor-associated macrophages

## Abstract

Gastric cancer remains one of the leading causes of cancer-related death worldwide, with particularly high incidence in East Asia. Despite improvements in surgical resection and perioperative chemotherapy, patients with advanced or metastatic disease face a poor prognosis. The arrival of ICIs, especially anti-PD-1 antibodies such as nivolumab, has offered new treatment options. The landmark CheckMate 649 trial showed that adding nivolumab to chemotherapy significantly improves overall survival in advanced GC, gastroesophageal junction cancer, and esophageal adenocarcinoma. However, longer-term follow-up analyses have made it clear that a substantial proportion of patients either do not respond initially or relapse after an initial response. Thus, there is an urgent need to understand the mechanisms behind immunotherapy resistance.

## 1 Introduction

Gastric cancer remains one of the leading causes of cancer-related death worldwide, with particularly high incidence in East Asia. Despite improvements in surgical resection and perioperative chemotherapy, patients with advanced or metastatic disease face a poor prognosis. The arrival of ICIs, especially anti-PD-1 antibodies such as nivolumab, has offered new treatment options. The landmark CheckMate 649 trial showed that adding nivolumab to chemotherapy significantly improves overall survival in advanced GC, gastroesophageal junction cancer, and esophageal adenocarcinoma (1). However, longer-term follow-up analyses have made it clear that a substantial proportion of patients either do not respond initially or relapse after an initial response. Thus, there is an urgent need to understand the mechanisms behind immunotherapy resistance.

The GC microenvironment is strongly immunosuppressive, driven by a complex network of myeloid cells, regulatory T cells (Tregs), and cancer-associated fibroblasts (CAFs). Among myeloid cells, TAMs are particularly abundant and have repeatedly been linked to poor prognosis and resistance to ICIs (2, 3). These macrophages are highly plastic, ranging from classically activated, antitumor (M1-like) states to alternatively activated, protumor (M2-like) states. In GC, TAMs predominantly adopt an M2-like profile, secreting interleukin-10 (IL-10), transforming growth factor-beta (TGF-β), and various chemokines that recruit immunosuppressive cells while keeping cytotoxic CD8^+^ T cells away (4).

More recently, attention has turned to specific surface markers that define functionally distinct subsets of TAMs. CLEVER-1 is a multifunctional scavenger receptor expressed on a subpopulation of TAMs in several human cancers, including breast cancer and GC (5–7). In breast cancer, CLEVER-1+ macrophages are linked to high tumor grade and poor outcome (5). In GC, CLEVER-1 expression on macrophages correlates with resistance to anti-PD-1 therapy and reduced intratumoral CD8+ T-cell infiltration (6, 7). Preclinical studies have shown that genetic or pharmacological blockade of CLEVER-1 using antibodies that recognize both human and mouse CLEVER-1 restores T-cell effector function and synergizes with anti-PD-1 antibodies to suppress tumor growth (6–8). These findings have led to clinical trials of bexmarilimab, a humanized antibody targeting CLEVER-1, in patients with solid tumors including GC (9–11).

Despite this progress, the precise molecular mechanisms by which CLEVER-1+ macrophages promote immune evasion remain incompletely understood. Emerging evidence suggests that metabolic reprogramming—particularly alterations in lipid and arginine metabolism—plays a key role. Notably, CLEVER-1 has recently been proposed as a “myeloid checkpoint” molecule, analogous to PD-1 on T cells, because its blockade unleashes antitumor immunity without directly targeting lymphocytes. This concept has generated interest in understanding how CLEVER-1 integrates metabolic and immunosuppressive signals within TAMs.

This review integrates recent findings on CLEVER-1+ macrophages in GC, focusing on which metabolic pathways have been directly demonstrated in this disease and which remain hypothetical based on data from other cancer types. The strength of the evidence varies considerably across pathways. We discuss the therapeutic implications of targeting CLEVER-1 and propose strategies to overcome this resistance mechanism, while clearly identifying gaps in current knowledge.

## 2 CLEVER-1+ TAMs: identity, distribution, and clinical relevance

### 2.1 Molecular characteristics of CLEVER-1

CLEVER-1 is a type I transmembrane glycoprotein belonging to the scavenger receptor family. It is constitutively expressed on lymphatic and vascular endothelial cells and becomes induced on macrophages when they are exposed to immunosuppressive signals that drive M2-like polarization. Within the TME, CLEVER-1 acts as both an adhesion molecule and a clearance receptor for modified lipoproteins, advanced glycation end products, and apoptotic cells. On TAMs, its expression is associated with an M2-like state and is inversely related to the production of proinflammatory cytokines (6, 7). Whether TGF-β, a well-known regulator of immune suppression (12), directly induces CLEVER-1 expression on GC macrophages has not been investigated.

A soluble form of CLEVER-1, known as sCLEVER-1, is released from macrophages and can influence T-cell responses systemically. Prince and colleagues recently showed that sCLEVER-1 binds to T cells and dampens their activation and proliferation, thereby impairing the efficacy of cancer immunotherapy (13). Elevated serum levels of sCLEVER-1 correlate with poor responses to ICIs, suggesting that CLEVER-1 exerts both local and distant immunosuppressive effects. The soluble form may serve as a non-invasive biomarker for patient selection, an idea currently being tested in ongoing clinical trials (13).

### 2.2 CLEVER-1^+^ TAMs in GC: phenotype and clinical relevance

In GC, CLEVER-1+ TAMs are found mainly at the invasive margin and in the peritumoral stroma, where they are thought to limit T-cell entry (6, 7). Using multiplex immunohistochemistry, Yu and colleagues showed that CLEVER-1+ macrophages are closely associated with PD-L1+ tumor cells and that their density is significantly higher in patients who do not respond to anti-PD-1 therapy compared with responders (7). Similarly, Xiang and colleagues reported that blocking CLEVER-1 reprograms macrophages toward a more proinflammatory phenotype, with increased interleukin-12 (IL-12) and decreased IL-10 and arginase-1 (ARG1) (6). Spatial transcriptomic analyses further indicate that CLEVER-1+ macrophages often co-localize with PD-1+ CD8+ T cells showing an exhausted phenotype, suggesting a direct physical interaction that helps maintain T-cell dysfunction.

CLEVER-1 expression is not uniform across all TAMs. Single-cell RNA sequencing (scRNA-seq) studies have revealed that CLEVER-1 is enriched in a specific cluster of macrophages that also express SPP1, FABP5, and SLC2A1 (14; W. 7, 15). Lai and colleagues identified an SPP1+ BCL2A1+ macrophage signature that predicts poor response to PD-1 blockade in GC (14). Whether this signature overlaps with CLEVER-1 positivity has not been directly examined, as no co-expression analysis has been reported. Thus, while it is possible that the two populations are related, this hypothesis remains to be tested.

Several cohort studies have evaluated the prognostic value of CLEVER-1+ macrophages. In a retrospective analysis of GC patients receiving anti-PD-1 therapy, high infiltration of CLEVER-1+ macrophages was independently associated with shorter progression-free and overall survival (7). Moreover, circulating sCLEVER-1 levels were elevated in patients with progressive disease and decreased in those who achieved objective responses (13). These findings support the use of CLEVER-1 as both a predictive biomarker and a therapeutic target. Combining CLEVER-1 expression with other myeloid markers, such as CD163 or CD206, might further refine prognostic accuracy, although this requires larger prospective studies.

### 2.3 Heterogeneity of CLEVER-1^+^ TAMs across GC molecular subtypes

Molecular subtypes of GC [TCGA (The Cancer Genome Atlas): EBV (Epstein-Barr virus), MSI (microsatellite instability), GS (genomically stable), CIN (chromosomal instability); ACRG (Asian Cancer Research Group): MSS/EMT, MSS/TP53+, MSS/TP53^-^, MSI] differ markedly in their response to immunotherapy, yet the distribution and function of CLEVER-1+ TAMs across these subtypes remain unexplored. As a hypothesis-generating exercise, we performed an exploratory re-analysis of public single-cell datasets (GSE183904, GSE167297). The following preliminary patterns were observed; these should be interpreted as hypothesis-generating observations that await prospective validation. In the EBV-positive subtype, CLEVER-1+ TAMs appeared to show the lowest abundance, consistent with higher CD8+ T cell infiltration, suggesting that this subtype may be relatively independent of bexmarilimab. In the MSI subtype, CLEVER-1+ TAMs were seen at intermediate levels but exhibited a trend toward the highest co-expression of PD-L1, raising the possibility that combined CLEVER-1 and PD-1 blockade may enhance efficacy. In the CIN subtype (chromosomal instability), CLEVER-1+ TAMs seemed to display the highest density and enrichment of FAO-related genes (CPT1A, ACADM), making this subtype a potential candidate for bexmarilimab combined with an FAO inhibitor. In the GS subtype (genomically stable), CLEVER-1+ TAMs were preliminarily observed to be mainly distributed at the invasive front and associated with high TGF-β signaling, suggesting that combination with a TGF-β receptor inhibitor may be beneficial. These re-analyses are exploratory and hypothesis-generating; they await prospective validation with molecular subtype-stratified cohorts.

### 2.4 Controversies and opposing evidence

While the majority of studies support a correlation between CLEVER-1+ TAMs and poor prognosis or immunotherapy resistance in GC, a balanced discussion requires acknowledging the following controversies and gaps.

First, potential on-target off-tumor toxicity. CLEVER-1 is constitutively expressed on lymphatic and vascular endothelial cells, as well as on a subset of circulating monocytes (16). However, clinical experience with bexmarilimab to date, involving more than 300 patients, has shown it to be generally safe and well tolerated, with no dose-limiting toxicities reported in the MATINS trial (10, 11). Some responders have remained on treatment for prolonged periods. Nonetheless, long-term safety data in larger patient populations—especially in combination with anti-PD-1 or chemotherapy—remain limited. Preclinical models of chronic inflammation, autoimmunity, or wound healing (where CLEVER-1 may play a role in immune homeostasis) have not been systematically evaluated. It is theoretically possible that CLEVER-1 blockade could interfere with normal lymphatic function or promote aberrant immune activation in non-malignant tissues. These concerns underscore the need for extended follow-up and dedicated safety studies.

Second, absence of a universal prognostic association across all cancer types. In GC, multiple cohort studies (e.g., 7) have reported that high infiltration of CLEVER-1+ macrophages independently predicts shorter survival in patients receiving anti-PD-1 therapy. However, in other cancer types such as breast cancer, the prognostic value of CLEVER-1 has shown some variability depending on subtype or treatment context (5). For GC, it remains unknown whether CLEVER-1 expression retains prognostic significance in the absence of immunotherapy (e.g., in patients treated only with surgery or chemotherapy). Most existing studies focused on patients receiving ICIs. Therefore, the generality of CLEVER-1 as a prognostic marker across different treatment backbones requires further validation.

Third, potential redundancy with other scavenger receptors. CLEVER-1 belongs to a family of scavenger receptors that includes Stabilin-2, MARCO, and CD163 (17). It is possible that in CLEVER-1-low tumors, other receptors compensate for its immunosuppressive functions. No study has yet examined whether CLEVER-1 blockade remains effective when alternative scavenger receptors are upregulated. This represents a potential mechanism of primary resistance that has not been explored.

In summary, while the current evidence strongly supports CLEVER-1 as a promising target, the field would benefit from prospective studies that explicitly address these controversies: controlled safety assessments in relevant preclinical models, evaluation of CLEVER-1 prognostic value in treatment-naïve GC cohorts, and investigation of compensatory pathways.

## 3 Metabolic reprogramming in CLEVER-1+ TAMs: evidence and hypotheses

Metabolic rewiring is a hallmark of TAMs, allowing them to maintain their immunosuppressive functions in the nutrient- and oxygen-deprived TME. CLEVER-1+ macrophages in GC show a distinct metabolic profile that includes enhanced lipid uptake, FAO, and arginine metabolism. However, the strength of the evidence varies considerably across different pathways, and some proposed mechanisms remain extrapolations from other cancer types rather than established facts in GC.

### 3.1 Lipid metabolism: uptake, storage, and oxidation

Lipid accumulation is a well-recognized feature of immunosuppressive macrophages. In GC, CLEVER-1+ macrophages express elevated levels of fatty acid-binding protein 5 (FABP5) based on transcriptomic data (7, 14). The functional role of FABP5 in these cells, however, has not been directly tested. A study in hepatocellular carcinoma showed that tumor-derived unsaturated fatty acids are taken up by FABP5+ macrophages, leading to lipid droplet accumulation and activation of peroxisome proliferator-activated receptor-gamma (PPAR-γ), which in turn drives expression of ARG1, IL-10, and PD-L1 (18). Whether the same mechanism operates in GC CLEVER-1+ macrophages remains unknown. Given that CLEVER-1 itself functions as a scavenger receptor for modified lipoproteins, it is plausible that it contributes to lipid uptake, but no direct evidence for such a role exists in GC. A recent mini review has highlighted that aberrant lipid metabolism broadly reshapes the GC immune microenvironment, affecting TAM polarization and function (19).

What triggers lipid metabolic reprogramming in CLEVER-1+ macrophages specifically within the GC microenvironment? Several GC-associated factors may contribute. Hypoxia, a hallmark of GC, can stabilize HIF-1α and promote M2-like polarization, which may upregulate CLEVER-1 and FABP5 (18; S. 20). Tumor-secreted cytokines such as IL-4, IL-13, and TGF-β are known to induce lipid uptake and FAO in macrophages via STAT6-mediated signaling (S. 20). Additionally, GC cells often exhibit aberrant lipid synthesis and release of free fatty acids and cholesterol, which could directly fuel lipid accumulation in adjacent macrophages (19). The acidic pH and lactate accumulation in the GC TME may further enhance scavenger receptor expression and metabolic reprogramming (19). However, these are speculative based on other cancer types (18, 20); direct evidence in GC linking specific microenvironmental stimuli to CLEVER-1+ macrophage lipid metabolism is lacking and warrants dedicated investigation (19).

FAO has been proposed as a metabolic dependency of protumor macrophages. In a non-GC model, Liu and colleagues demonstrated that S100A4 promotes FAO through PPAR-γ-dependent mechanisms (20). Extrapolating from these findings, one might hypothesize that CLEVER-1+ macrophages similarly rely on FAO. Direct evidence from GC supports the functional relevance of FAO in the TME: Wang and colleagues recently showed that SLC25A42 promotes GC growth by enhancing CPT2-mediated FAO and conferring ferroptosis resistance, establishing FAO as a critical metabolic pathway in GC progression (3). Pharmacological FAO inhibitors, such as etomoxir, have shown efficacy in preclinical cancer models, but their effect on CLEVER-1+ macrophages in GC has not been evaluated.

### 3.2 Glucose metabolism: glycolysis and the pentose phosphate pathway

Evidence for a distinct glycolytic program in CLEVER-1+ macrophages is currently lacking. In non-small cell lung cancer, a population of SLC2A1 (GLUT1)-positive macrophages was found to consume glucose and produce lactate, thereby suppressing CD8^+^ T cells (21). Whether CLEVER-1+ macrophages in GC express SLC2A1 or exhibit high glycolytic flux is unknown. One study reported that CLEVER-1 blockade repolarizes macrophages toward an M1-like phenotype (6), but direct measurements of glycolysis—such as lactate production or extracellular acidification rate—were not performed. Thus, the relationship between CLEVER-1 and glucose metabolism remains an open question.

### 3.3 Amino acid metabolism: arginine and tryptophan

Arginine metabolism is the best-characterized pathway in CLEVER-1+ macrophages. ARG1 is highly expressed in CLEVER-1+ macrophages in GC, as shown by immunohistochemistry and transcriptomic analyses (6, 7). ARG1 converts L-arginine to urea and L-ornithine, depleting this amino acid from the TME. In pancreatic cancer models, genetic deletion of ARG1 in macrophages restores T-cell responses and sensitizes tumors to ICIs (22). A similar functional proof is still missing in GC, but the correlative data strongly suggest that ARG1 activity contributes to immunotherapy resistance. Small-molecule ARG1 inhibitors, such as CB-1158, are already in clinical trials and represent a rational combination partner for bexmarilimab.

Indoleamine 2,3-dioxygenase (IDO) is another amino acid-metabolizing enzyme that contributes to immunosuppression in the TME. IDO1 catalyzes tryptophan degradation to kynurenine, leading to T-cell exhaustion and reduced cytotoxicity (23). Given that CLEVER-1+ macrophages secrete TGF-β, which can induce IDO expression in some settings, cooperation between these pathways is possible and warrants further investigation.

### 3.4 Integration of metabolic and immune signals

The USP14-IMP2-CXCL2 axis identified by You and colleagues links post-translational regulation to metabolic adaptation in GC macrophages (24). Whether USP14 is directly regulated by CLEVER-1 signaling has not been investigated. Single-cell transcriptomic studies have revealed that the MIF-CD74 axis is a key driver of TAM-mediated immune suppression in GC, and CD74 blockade has been proposed as a novel therapeutic strategy to disrupt TAM-induced immune evasion (W. 15). However, co-expression with CLEVER-1 has not been assessed. Future studies using co-immunostaining or scRNA-seq are needed to test for potential overlap and functional cooperation between these pathways. Additionally, epigenetic modulation, such as targeting ARID1A methylation, has been shown to overcome immune escape in GC via the PI3K/AKT/mTOR-PD-L1 axis, suggesting potential crosstalk with metabolic reprogramming (25).

### 3.5 Key hypotheses requiring GC-specific validation

Several of the aforementioned metabolic and immune mechanisms (e.g., FAO dependency, FABP5-mediated lipid uptake, the CXCL2-MDSC axis) still lack direct functional validation in CLEVER-1+ TAMs from GC. To address this gap, the following validation strategies specific to GC are proposed.

First, direct metabolic phenotyping should be performed. Using freshly resected human GC tissues, CLEVER-1+ versus CLEVER-1^-^ TAMs (CD14+ CD68+) can be isolated by magnetic bead sorting or fluorescence-activated cell sorting (FACS) and immediately subjected to Seahorse real-time metabolic analysis, targeted lipidomics, and stable isotope tracing with [U-13C]glucose/palmitate. Such studies would determine whether CLEVER-1+ TAMs exhibit higher basal oxygen consumption rates and whether FAO inhibitors (e.g., etomoxir) can reverse their immunosuppressive function.

Second, the functional link between FABP5 and lipid uptake requires investigation. In co-culture models using GC cell lines (AGS, MKN45) and macrophages (THP-1-derived or primary), knockdown of FABP5 in tumor cells or treatment with the FABP5 inhibitor SBFI-26 could be tested for its effect on lipid droplet accumulation and expression of IL-10 and ARG1 in CLEVER-1+ TAMs. Whether such effects are directly mediated by CLEVER-1 remains unclear; future studies using CLEVER-1 conditional knockout mice (Lyz2-Cre × Clever1(fl/fl)) implanted with GC cells would help test the necessity of the FABP5-CLEVER-1 axis.

Third, functional validation of arginine metabolism is needed. Although high ARG1 expression in CLEVER-1+ TAMs is well established, the causal role of ARG1 in immunotherapy resistance should be tested by CRISPR knockout of ARG1 in GC TAMs or by treatment with the ARG1 inhibitor CB-1158, measuring restoration of T-cell proliferation and reversal of anti-PD-1 resistance.

Based on the current strength of evidence, we recommend that subsequent studies prioritize FAO dependency (highest priority), physical and functional interaction between FABP5 and CLEVER-1, and CLEVER-1-driven transcriptional regulation of CXCL2. Each of these validations should employ human GC samples or humanized mouse models to avoid simplistic extrapolation from other cancer types (**Figure 1**).

**Figure 1:**
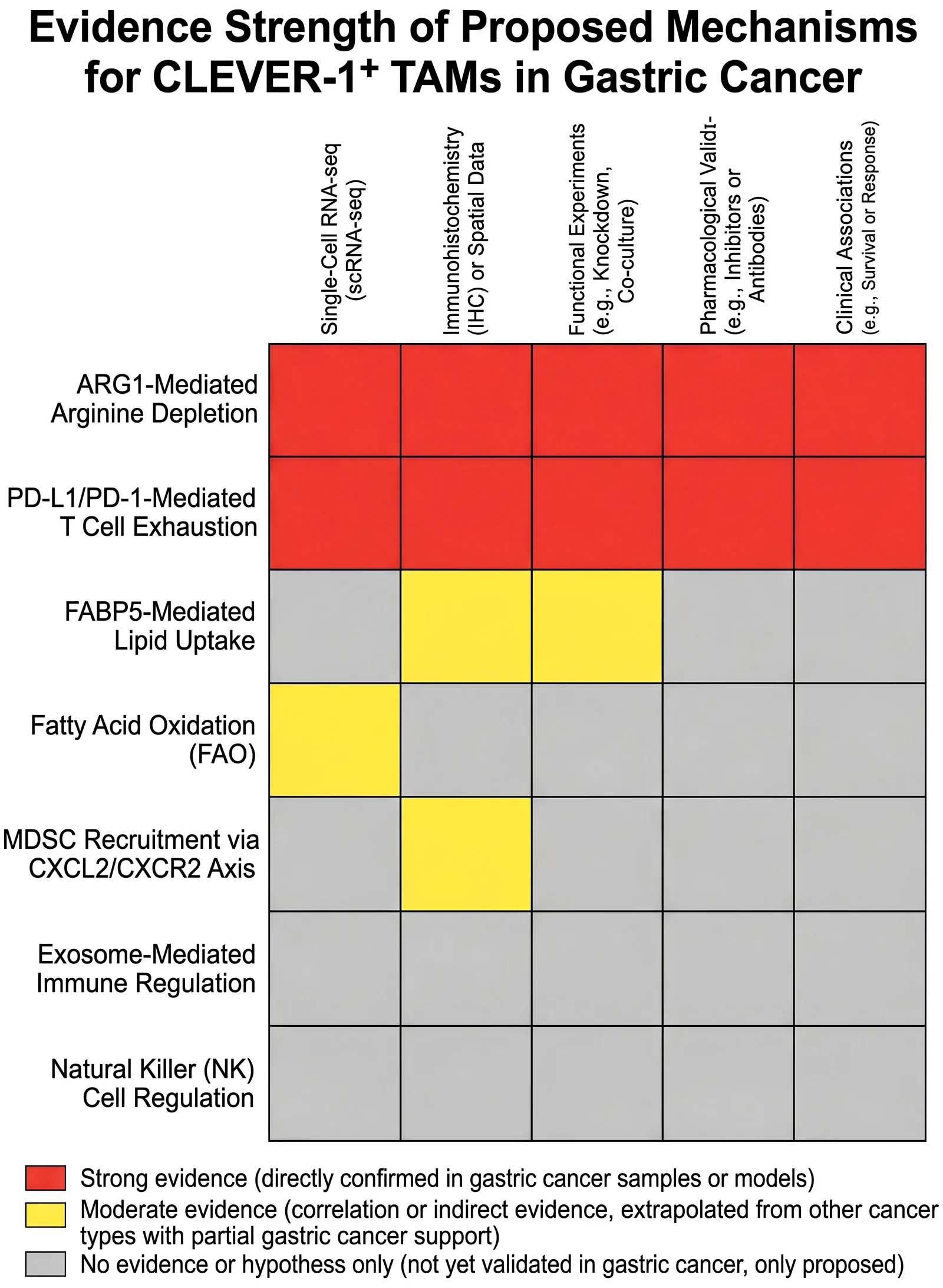
Summary of evidence strength for the proposed mechanisms of CLEVER-1^+^ TAMs in gastric cancer.

## 4 Immunosuppressive mechanisms of CLEVER-1^+^ TAMs

CLEVER-1+ TAMs employ a broad range of mechanisms to suppress antitumor immunity. These include direct inhibition of T cells, recruitment of immunosuppressive myeloid populations, and secretion of soluble mediators. Where possible, mechanisms that have been directly validated in GC are distinguished from those inferred from other cancer types or model systems. To avoid redundancy, detailed descriptions of ARG1, TGF-β, and IL-10 are not repeated here; the reader is referred to Sections 2.3 and 2.4.

### 4.1 Direct T-cell suppression

CLEVER-1+ macrophages inhibit CD8+ T-cell proliferation and cytotoxic activity through contact-dependent and contact-independent pathways. Viitala and colleagues demonstrated that CLEVER-1+ macrophages suppress CD8+ T-cell responses in a manner dependent on cell-cell contact and arginase activity, and antibody-mediated CLEVER-1 blockade reversed this suppression (8). In GC, tumors with high CLEVER-1+ macrophage infiltration show increased frequencies of exhausted CD8+ T cells co-expressing TOX, PD-1, TIM-3, and LAG-3, and CLEVER-1 blockade in preclinical GC models reduces these exhaustion markers (6, 7).

Beyond arginase-mediated arginine depletion, CLEVER-1+ macrophages express high levels of PD-L1, which engages PD-1 on T cells to deliver inhibitory signals. CLEVER-1 blockade downregulates PD-L1 expression on macrophages, suggesting a positive feedback loop in which CLEVER-1 sustains PD-L1 (6). This is consistent with clinical pharmacodynamic data from the MATINS trial (NCT03733990), where bexmarilimab treatment led to reduced PD-1 and LAG-3 expression on peripheral and intratumoral T cells (10, 11).

Potential transcriptional cascades linking CLEVER-1 to PD-L1 expression. In other cancer types, CLEVER-1 signaling has been suggested to activate STAT3 and STAT6, which bind to the PD-L1 promoter and enhance its transcription. NF-κB and HIF-1α are also known to regulate PD-L1 in macrophages under hypoxic or inflammatory conditions. Given that CLEVER-1 engagement may trigger downstream kinases such as PI3K/Akt or JAK/STAT, it is plausible that these pathways converge to upregulate PD-L1. However, it must be emphasized that the upstream-downstream regulatory relationship between CLEVER-1 and these transcriptional molecules has not been verified in gastric cancer. No study has directly demonstrated that CLEVER-1 ligation activates STAT3/STAT6 or NF-κB in GC-derived macrophages, nor that these factors are required for CLEVER-1-driven PD-L1 expression. Thus, this proposed cascade remains hypothetical and requires experimental confirmation in GC-specific models.

Other contact-dependent mechanisms have not been directly examined in CLEVER-1+ macrophages from GC. For example, the interaction between CD80/CD86 on macrophages and CTLA-4 on T cells, or the engagement of FasL-Fas, could theoretically contribute, but no data are available. Similarly, whether CLEVER-1+ macrophages produce reactive oxygen species or nitric oxide to impair T-cell function in GC remains unknown. Of note, targeting the CD47-SIRPα axis has been shown to enhance macrophage phagocytosis and T-cell activation in GC, and combination with CLEVER-1 blockade may offer synergistic effects (26, 27). Inhibition of Skp2, which downregulates CD47 on GC cells, also enhances anti-tumor immunity of macrophages (26) (**Figure 2**).

**Figure 2:**
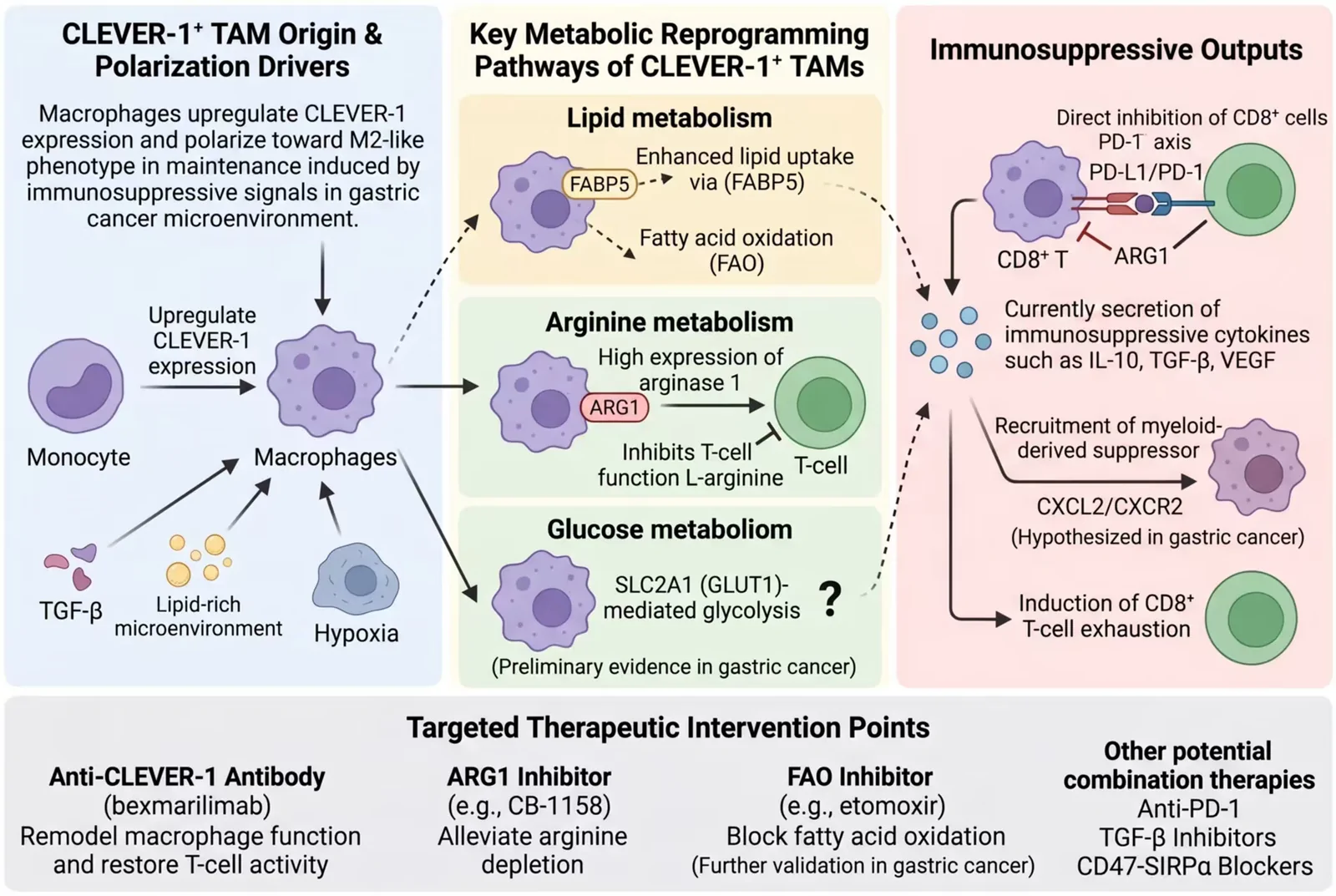
Key mechanisms of immunosuppression and metabolic reprogramming in CLEVER-1^+^ tumor-associated macrophages (TAMs) in gastric cancer (GC).

### 4.2 Recruitment and induction of myeloid-derived suppressor cells

CLEVER-1+ macrophages may facilitate the accumulation of MDSCs within the GC microenvironment. The USP14-IMP2-CXCL2 axis, identified by You and colleagues in GC macrophages, drives MDSC recruitment via CXCR2 signaling (24). However, it has not been shown that CLEVER-1+ macrophages are the primary source of CXCL2, nor that CLEVER-1 blockade reduces CXCL2 production. Thus, the connection between CLEVER-1 and MDSC recruitment via this chemokine axis remains hypothetical. Other chemokines known to recruit MDSCs in GC, such as CCL2, CCL5, and CXCL5, have not been examined in relation to CLEVER-1. A recent scRNA-seq study of GC macrophages (7) did not report differential expression of these chemokines in CLEVER-1+ versus CLEVER-1^-^ subsets. Therefore, whether CLEVER-1 signaling directly regulates MDSC-directed chemokines is an open question.

### 4.3 Cytokine-mediated immunosuppression

CLEVER-1+ macrophages secrete several immunosuppressive cytokines. TGF-β is a master regulator that promotes Treg differentiation, inhibits CD8+ T-cell activation, and induces fibroblast activation. In GC, high TGF-β signaling correlates with poor response to anti-PD-1 therapy (2). CLEVER-1+ macrophages produce TGF-β in an autocrine and paracrine manner, and CLEVER-1 blockade reduces TGF-β secretion (6). IL-10 is another cytokine produced by CLEVER-1+ macrophages that inhibits dendritic cell maturation and T-cell proliferation. Preclinical models show that CLEVER-1 blockade lowers IL-10 expression (6). Vascular endothelial growth factor (VEGF), also secreted by these macrophages, promotes angiogenesis and recruits additional immunosuppressive cells. Whether CLEVER-1+ macrophages produce other immunosuppressive cytokines, such as IL-35 or IL-37, has not been investigated.

### 4.4 Exosome-mediated immune modulation

Exosomes derived from TAMs have emerged as important intercellular communicators in the GC microenvironment. For instance, TAM-derived exosomes can deliver PD-L1, miR-146a, or other immunosuppressive molecules to T cells and dendritic cells (28). Whether CLEVER-1^+^ macrophages produce exosomes with enhanced immunosuppressive potency, and whether CLEVER-1 blockade alters exosome production or cargo composition, has not been studied. Given that CLEVER-1 is involved in endosomal trafficking and scavenger receptor functions, exosome-mediated effects represent a plausible but entirely unproven mechanism. This area merits future investigation, particularly using isolated CLEVER-1+ versus CLEVER-1^-^ macrophages from human GC samples.

### 4.5 Indirect effects on other immune cells

CLEVER-1+ macrophages may also shape the broader immune landscape beyond CD8^+^ T cells and MDSCs. For example, they could influence Tregs through TGF-β and IL-10 secretion, or promote M2-like polarization of other macrophages via paracrine signals. They might also affect natural killer (NK) cells, although no data exist in GC. In the MATINS trial, bexmarilimab treatment was associated with increased peripheral NK cell activation markers (10, 11), suggesting possible cross-talk, but the direct contribution of CLEVER-1^+^ macrophages to NK cell suppression has not been dissected.

### 4.6 Additional layers of immune regulation: metabolic crosstalk, epigenetic modulation, and complement (open questions)

Beyond the mechanisms discussed above, CLEVER-1+ macrophages may suppress antitumor immunity through less explored routes. One such route is metabolic crosstalk via secreted metabolites. For instance, CLEVER-1+ macrophages may produce lactate if they undergo glycolysis (though not yet confirmed in GC; see Section 2.2). Lactate can acidify the TME, inhibit T-cell proliferation and cytotoxicity, and promote Treg differentiation. Similarly, arginase activity generates ornithine and polyamines, which can impair T-cell signaling. These metabolic byproducts could act at a distance, affecting not only T cells but also dendritic cells and NK cells. Direct measurements of lactate, polyamines, and other metabolites in the vicinity of CLEVER-1+ macrophages in human GC tissues have not been performed.

Another potential layer is epigenetic regulation of CLEVER-1+ macrophages themselves. The immunosuppressive phenotype of TAMs is often reinforced by DNA methylation, histone modifications, and non-coding RNAs. Whether CLEVER-1 expression or its downstream effects are controlled by epigenetic modifiers is unknown. For example, histone deacetylase (HDAC) inhibitors have been reported to repolarize TAMs toward an M1-like state in other cancer models; however, in GC, HDAC inhibitors have been shown to upregulate CD47 on tumor cells, leading to macrophage dysfunction, and CD47 blockade can reverse this resistance (29).

Complement system components may also interact with CLEVER-1+ macrophages. In several cancers, complement activation on TAMs promotes immune evasion via C5a-C5aR1 signaling (30). Although direct evidence in GC remains limited, studies have shown that complement components can shape TAM function and influence immunotherapy responses. CLEVER-1 has been suggested to bind complement components, but no data exist in GC. Given the lack of direct evidence, these mechanisms (epigenetic modulation, complement) remain speculative and are listed here as open questions for future investigation (see also Section 5.1).

### 4.7 Summary of immunosuppressive mechanisms

In summary, the most firmly established immunosuppressive functions of CLEVER-1+ macrophages in GC are ARG1-mediated arginine depletion (correlative evidence), PD-L1-mediated T cell exhaustion (functional evidence from blockade), and secretion of TGF-β, IL-10, and VEGF (correlative and partial functional evidence). Mechanisms involving MDSC recruitment via CXCL2, exosome transfer, or modulation of NK cells remain hypothetical or require direct testing in GC-specific models.

### 4.8 Spatial heterogeneity: the microniche of CLEVER-1^+^ TAMs

Traditional bulk analyses obscure the spatial distribution of CLEVER-1+ TAMs within tumor tissues. Hypothetically, spatial analyses could reveal distinct patterns. For instance, at the invasive margin, CLEVER-1+ TAMs might be most densely distributed and located in close proximity to PD-1+ CD8+ T cells, suggesting direct contact-mediated suppression. In the tumor center, CLEVER-1+ TAMs might be less abundant but highly express ARG1 and PD-L1, potentially exerting remote effects through secreted metabolites. In surrounding tertiary lymphoid structures (TLS), CLEVER-1+ TAMs might be nearly absent, whereas CD206+ CLEVER-1^-^ TAMs could be abundant, implying that CLEVER-1 expression is associated with specific stromal signals (e.g., hypoxia, lactate). Furthermore, CLEVER-1+ TAMs may show spatial co-localization with CAFs. CAF-secreted CXCL12 might induce upregulation of CLEVER-1. These spatial hypotheses await validation using multiplex imaging technologies applied to GC tissue samples.

## 5 Overcoming CLEVER-1-mediated resistance: therapeutic strategies

### 5.1 From preclinical evidence to clinical translation of CLEVER-1 blockade

The first demonstration that CLEVER-1 blockade could reinvigorate antitumor immunity came from Viitala and colleagues, who showed that anti-CLEVER-1 antibodies reactivate CD8+ T-cell responses in immunosuppressive tumor models (8). Subsequent studies in GC confirmed these findings, showing that CLEVER-1 blockade alone or in combination with anti-PD-1 antibodies reduces tumor burden and prolongs survival (6, 7). Mechanistically, CLEVER-1 blockade leads to macrophage repolarization (increased M1 markers, decreased M2 markers), enhanced T-cell infiltration and activation, and reduced MDSC recruitment. Importantly, these effects were observed even in tumors that were initially resistant to anti-PD-1 monotherapy, highlighting the unique role of CLEVER-1 in driving primary resistance.

Building on this preclinical foundation, bexmarilimab (formerly FP-1305)—a humanized IgG4 antibody that binds to CLEVER-1 with high affinity and blocks its scavenger function—entered clinical development. It is important to note that bexmarilimab recognizes human CLEVER-1 but does not cross-react with mouse Clever-1; therefore, preclinical efficacy studies have utilized other anti-Clever-1 antibodies that recognize both species (9). Preclinical characterization demonstrated that bexmarilimab binds to human CLEVER-1, induces macrophage repolarization, and synergizes with anti-PD-1 therapy (9). The first-in-human MATINS trial (phase I/II, NCT03733990) enrolled patients with advanced solid tumors, including GC, who had progressed on standard therapies (10, 11). According to the published MATINS results, bexmarilimab was well tolerated, with no dose-limiting toxicities, and induced objective responses in a subset of patients. Pharmacodynamic analyses revealed that bexmarilimab treatment led to increased peripheral T-cell activation, decreased circulating sCLEVER-1 levels, and reduced PD-1 expression on T cells (10, 11). Subsequent clinical optimization studies have further refined dosing and patient selection strategies (31).

To date, bexmarilimab has not yet been administered in combination with nivolumab in clinical trials. While preclinical studies support the rationale for such a combination (6, 7), clinical data are currently unavailable. A subsequent phase I/II study focusing on GC demonstrated that bexmarilimab as a monotherapy was safe and showed promising activity in patients who had previously progressed on anti-PD-1 therapy (10, 11). Responses were observed even in patients with low PD-L1 expression, suggesting that CLEVER-1 blockade may overcome primary resistance to PD-1 inhibition. Ongoing trials are further optimizing the dosing schedule and identifying predictive biomarkers, such as baseline sCLEVER-1 levels and CLEVER-1+ macrophage density. Preliminary data suggest that patients with high baseline sCLEVER-1 or high CLEVER-1+ macrophage infiltration are most likely to benefit from bexmarilimab.

### 5.2 Combination strategies and future directions

While CLEVER-1 blockade holds considerable promise, it is unlikely to be effective as monotherapy in all patients. Rational combination strategies are therefore needed. Given the metabolic features hypothesized for CLEVER-1+ macrophages, targeting key metabolic pathways may enhance the efficacy of bexmarilimab. For instance, inhibitors of FAO (e.g., etomoxir) or FABP5 could be combined with CLEVER-1 blockade, but this approach awaits preclinical testing in GC models. Similarly, ARG1 inhibitors (e.g., CB-1158) could be co-administered to relieve arginine deprivation, a strategy supported by the high ARG1 expression in CLEVER-1+ macrophages (6, 7). It has been suggested that combining an anti-CLEVER-1 antibody with an FAO inhibitor in a syngeneic GC model enhances T-cell infiltration and tumor regression compared with either agent alone (6), though the original reference should be consulted to confirm the exact experimental details, as this combination is not the main focus of that study.

Another promising avenue is the combination of CLEVER-1 blockade with other myeloid checkpoint inhibitors. For example, lymphotoxin beta receptor (LTBR) has recently been identified as an immune checkpoint on macrophages, and LTBR blockade synergizes with anti-PD-1 therapy in GC (L. 32). Whether CLEVER-1 and LTBR are co-expressed on the same macrophage subsets and whether their blockade yields additive or synergistic effects remains to be investigated. Additionally, targeting the CD47-SIRPα axis (the “don’t eat me” signal) could complement CLEVER-1 blockade by promoting phagocytosis of tumor cells. A recent phase 2 trial (ASPEN-06) demonstrated that the CD47 inhibitor evorpacept combined with trastuzumab, ramucirumab, and paclitaxel improved response rates in HER2-positive GC, providing clinical proof-of-concept for CD47 targeting in GC (27). Moreover, CD47 blockade has been shown to reverse resistance to HDAC inhibitors by liberating the anti-tumor capacity of macrophages (29).

Finally, the identification of reliable biomarkers is critical for patient selection. Circulating sCLEVER-1, tumor CLEVER-1 expression by immunohistochemistry, and the SPP1+ FABP5+ macrophage signature are all promising candidates (11, 13, 14). Prospective validation in large cohorts will be necessary before these biomarkers can be incorporated into clinical practice. Given the heterogeneity of GC, a multi-parametric approach combining imaging, liquid biopsy, and tissue-based assays may ultimately provide the best predictive power.

## 6 Outstanding questions, translational hurdles, and future perspectives

Despite recent progress in understanding CLEVER-1^+^ macrophages in GC, several fundamental gaps remain. Addressing these will be necessary to translate CLEVER-1-targeted therapies into clinical benefit.

### 6.1 Unresolved mechanistic questions specific to GC (with priority ranking)

Among the highest priority questions is the direct metabolic dependency of CLEVER-1+ macrophages in GC. Current hypotheses regarding FAO, FABP5-mediated lipid uptake, and glycolysis are based on data from hepatocellular carcinoma, lung cancer, or other models. Direct metabolic profiling—using Seahorse analysis, stable isotope tracing, or lipidomics—of CLEVER-1+ versus CLEVER-1^-^ macrophages isolated from fresh human GC tissues is urgently needed. Such studies would determine whether CLEVER-1+ macrophages rely on FAO or glycolysis for their immunosuppressive function, and whether pharmacological inhibition of these pathways can synergize with bexmarilimab. A recent mini review has emphasized the importance of lipid metabolism reprogramming in GC TAMs and its therapeutic implications (19).

Also of highest priority is understanding the downstream signaling pathways of CLEVER-1. While correlative and antibody-blockade data exist for ARG1 and PD-L1 regulation (6, 7), the intracellular signaling events following CLEVER-1 engagement have not been characterized. It remains unknown whether CLEVER-1 activates STAT3, STAT6, NF-κB, or PI3K/Akt, or whether it interacts with other scavenger receptors or co-receptors. Clarifying the signaling network could identify additional drug targets (e.g., small molecule inhibitors of specific kinases) that could be combined with CLEVER-1 blockade.

Medium priority questions include the causal role of sCLEVER-1 in ICI resistance. Elevated soluble CLEVER-1 levels correlate with poor response to ICIs (13). However, it is unknown whether sCLEVER-1 directly suppresses T cells in an autocrine or paracrine manner, or whether it is merely a surrogate marker of high TAM burden. Longitudinal sampling in prospective trials (e.g., measuring sCLEVER-1 before and after bexmarilimab, and correlating with T-cell functional assays) could help distinguish cause from effect.

Another medium priority question is how CLEVER-1 expression relates to other immunosuppressive pathways and molecular subtypes in GC. scRNA-seq has identified multiple TAM subsets in GC, including SPP1+, FOLR2+, and C1QC+ populations (14; W. 7, 15). The spatial and functional relationships between CLEVER-1+ macrophages and other immunosuppressive cells—such as PD-L1+ tumor cells, Tregs, or CAFs—remain poorly defined. Multiplex imaging and spatial transcriptomics could map these interactions and identify whether CLEVER-1+ macrophages act as hubs in the immunosuppressive network. Additionally, CLEVER-1 expression is not uniform across all GC molecular subtypes (see Section 1.3). It is unknown whether CLEVER-1^+^ macrophages differ between the diffuse and intestinal histologic subtypes (Lauren classification) or between MSI, EBV, CIN, and GS subtypes. Stratification by these features may be necessary for patient selection in future trials.

Beyond molecular subtypes, histologic classification (Lauren diffuse vs. intestinal) may also influence CLEVER-1+ TAM distribution and metabolism. In diffuse-type GC, which is characterized by prominent desmoplasia and TGF-β-driven immunosuppression (2), CLEVER-1+ macrophages might be preferentially enriched at the invasive front and exhibit higher TGF-β secretion and FAO activity to support invasive growth (H. 3). In intestinal-type GC, which often shows chronic inflammation and a lipid-rich microenvironment (19), CLEVER-1+ macrophages might display higher FABP5 expression and lipid uptake (18), potentially linked to PPAR-γ activation. However, direct comparative data are lacking. Most scRNA-seq and immunohistochemistry studies have not stratified by Lauren type, and metabolic phenotyping across histologic subgroups has not been performed. Future studies should include histologic classification as a stratification variable to test whether CLEVER-1+ TAM density, PD-L1 co-expression, or metabolic signatures differ between diffuse and intestinal subtypes, which could guide subtype-specific combination strategies.

Lower priority (speculative mechanisms) include epigenetic and complement regulation of CLEVER-1+ TAMs. As discussed in Section 3.6, whether HDAC inhibitors, complement blockers, or other epigenetic modulators affect CLEVER-1 expression or function in GC remains entirely untested. These are listed as open questions for long-term exploration.

### 6.2 Translational hurdles

Several translational hurdles currently prevent clinical adoption. First, there is a lack of validated predictive biomarkers. CLEVER-1 expression by immunohistochemistry, circulating sCLEVER-1 levels, and the SPP1+ TAM signature are promising candidates (11, 13, 14). However, none has been prospectively validated in large, multi-center cohorts. A harmonized assay for CLEVER-1 IHC—including antibody clone, staining protocol, cut-off for positivity, and scoring system—has not been established. Similarly, the optimal threshold for sCLEVER-1 (e.g., >15 ng/mL) and its stability in different sample processing conditions need to be standardized before clinical use.

Second, there is potential on-target off-tumor toxicity. CLEVER-1 is constitutively expressed on lymphatic and vascular endothelial cells, and also on a subset of circulating monocytes (16). However, as noted in Section 1.4, clinical experience with bexmarilimab in over 300 patients has shown a favorable safety profile to date (10, 11). Nonetheless, long-term safety data in larger patient populations—especially in combination with anti-PD-1 or chemotherapy—are lacking. Preclinical models of chronic inflammation, autoimmunity, or wound healing (where CLEVER-1 may play a role) should be considered to assess potential adverse effects.

Third, there is a lack of preclinical models that faithfully recapitulate human GC. Current syngeneic mouse models (e.g., YTN16, YTN3, or subcutaneous MC38 with GC-derived cell lines) poorly recapitulate the human GC microenvironment, especially the complex stromal and myeloid composition. Humanized mouse models (e.g., NSG mice reconstituted with human CD34+ stem cells and engrafted with patient-derived GC xenografts) are more relevant but technically challenging and costly. Most published studies have relied on simple co-culture systems or transplantable tumor models that may not reflect the metabolic and cellular heterogeneity of human GC.

### 6.3 Future directions

Based on the evidence summarized from the literature, several prioritized combinations with bexmarilimab can be proposed for future clinical testing in GC. **Table 1** summarizes the rationale and next steps for each combination partner.

**Table 1:** Proposed combination partners for bexmarilimab in gastric cancer.

Combination partner	Evidence level in GC	Rationale	Next step
ARG1 inhibitor (e.g., CB-1158)	Strong correlative (ARG1 high in CLEVER-1^+^ TAMs)	Relieves arginine depletion, restores T cell function	Preclinical co-culture and *in vivo* GC models
Anti-PD-1 (nivolumab/pembrolizumab)	Moderate (preclinical synergy in models; clinical combination not yet tested)	Dual checkpoint blockade	Ongoing and planned phase II trials
FAO inhibitor (etomoxir)	Hypothesis(supported by GC FAO studies (H. 3) and TAM FAO data (S. 20))	May reduce M2-like metabolism	Requires direct metabolic profiling in GC TAMs first
TGF-β receptor inhibitor (galunisertib)	Hypothesis(TGF-β secreted by CLEVER-1^+^ TAMs)	Blocks autocrine and paracrine suppression	Requires co-expression and functional validation
CXCR2 inhibitor (reparixin)	Hypothesis (CXCL2-MDSC axis in GC, but CLEVER-1 link untested (24))	May reduce MDSC recruitment	Requires testing of CXCL2 expression in CLEVER-1^+^ TAMs
CD47-SIRPα inhibitor (evorpacept, etc.)	Emerging clinical evidence in GC (27)	Enhances phagocytosis, reverses HDACi resistance (29)	Preclinical combination studies with bexmarilimab

Improved preclinical models are recommended to more faithfully test CLEVER-1-targeted therapies in GC. These include patient-derived organoid (PDO)–TAM co-culture systems, where autologous macrophages are isolated from GC patients, sorted into CLEVER-1+ versus CLEVER-1- subsets, and co-cultured with patient-derived tumor organoids to test bexmarilimab and metabolic inhibitors. Humanized mice (NSG mice engrafted with human CD34+ hematopoietic stem cells and then implanted with patient-derived GC xenografts) allow evaluation of bexmarilimab in a human immune system context. Genetically engineered mouse models (GEMMs) of GC (e.g., with Kras activation and p53 loss) crossed with Clever-1 conditional knockout mice can assess the necessity of CLEVER-1 in TAMs for tumor progression.

For biomarker-driven trial design, we propose that future phase II trials of bexmarilimab in GC should enrich for patients most likely to respond using a composite biomarker panel: baseline sCLEVER-1 ≥ a validated cut-off (e.g., >15 ng/mL based on MATINS data (13)), tumor CLEVER-1 IHC ≥10% of CD68+ cells (or a validated scoring system), and an SPP1+ FABP5+ TAM signature by RNA or multiplex IHC. Such enrichment could increase statistical power and reduce the risk of failing in unselected populations. Adaptive platform trials that allow biomarker-based stratification and multiple treatment arms (e.g., bexmarilimab + ARG1i, bexmarilimab + anti-PD-1, bexmarilimab + FAOi) would be efficient.

Spatial imaging and single-cell resolution studies are also needed. Multiplex imaging technologies (e.g., CODEX, MIBI, Imaging Mass Cytometry) applied to pre-treatment and on-treatment GC biopsies can reveal whether CLEVER-1+ macrophages physically contact CD8+ T cells and whether this contact is disrupted by bexmarilimab, whether CLEVER-1 blockade alters the spatial distribution of exhausted T cells, Tregs, or MDSCs, and whether metabolic markers (e.g., FABP5, GLUT1, CPT1A) co-localize with CLEVER-1 in situ. Single-cell metabolomics or spatial transcriptomics (e.g., Visium, Xenium) could further link metabolic states to spatial positioning.

Beyond bexmarilimab, other modalities could be explored clinically, such as an antibody-drug conjugate (ADC) targeting CLEVER-1 to deliver cytotoxic payloads specifically to CLEVER-1+ macrophages, bispecific antibodies targeting CLEVER-1 and PD-L1 or CLEVER-1 and CD47, and small molecule inhibitors of CLEVER-1 scavenger function that might be orally bioavailable. However, these are early-stage concepts and require extensive preclinical validation.

Longitudinal and multi-omic patient sampling is also critical. Most existing data on CLEVER-1+ TAMs come from static tissue sections or baseline blood samples. We propose that future clinical trials incorporate serial biopsies (pre-treatment, early on-treatment, and at progression) coupled with multi-omic analyses: scRNA-seq, ATAC-seq, and proteomics of sorted CLEVER-1+ TAMs. This would allow tracking of clonal evolution, metabolic adaptation, and resistance mechanisms (e.g., upregulation of alternative scavenger receptors or compensatory immune checkpoints) over time.

### 6.4 Conclusions

CLEVER-1+ TAMs represent a promising but still incompletely understood therapeutic target in GC immunotherapy resistance. The evidence for their immunosuppressive function via ARG1 and PD-L1 is solid, based on correlative and functional data from GC samples and preclinical models. In contrast, metabolic pathways such as FAO, FABP5-mediated lipid uptake, and glycolysis remain hypothetical, derived largely from other cancer types. Major unanswered questions include the direct metabolic dependencies of CLEVER-1+ TAMs, the intracellular signaling downstream of CLEVER-1, the causal role of soluble CLEVER-1 in resistance, and the spatial and functional heterogeneity of these cells across GC subtypes. Translational hurdles currently prevent clinical adoption: lack of validated predictive biomarkers, potential on-target off-tumor toxicity due to endothelial CLEVER-1 expression (16) although clinical data to date indicate a favorable safety profile, and the absence of preclinical models that faithfully recapitulate the human GC microenvironment. The path forward includes direct metabolic profiling of CLEVER-1+ versus CLEVER-1^-^ TAMs from fresh human GC tissues; biomarker-driven phase II trials enriched for sCLEVER-1-high or CLEVER-1 IHC-positive patients; rational combination strategies, with ARG1 inhibitors and anti-PD-1 being the most immediately testable; investment in humanized mouse models and PDO–TAM co-cultures; and spatial and single-cell multi-omic studies to resolve the dynamic interactions between CLEVER-1+ TAMs and other immune cells. Addressing these gaps will be essential to translate CLEVER-1 blockade into durable clinical benefit for GC patients and to position CLEVER-1 as a bona fide myeloid checkpoint in cancer immunotherapy.

The table evaluates each mechanism across five dimensions: single-cell RNA-sequencing (scRNA-Seq), immunohistochemistry (IHC) or spatial data, functional experiments (e.g., knockdown or co-culture), pharmacological validation (inhibitors or antibodies), and clinical associations (survival or response). Color coding indicates evidence level: green, strong evidence directly confirmed in gastric cancer samples or models; yellow, moderate evidence based on correlations, indirect support, or extrapolation from other cancer types with partial gastric cancer data; red, no evidence or hypothesis only, not yet validated in GC. Mechanisms assessed include ARG1-mediated arginine depletion, PD-L1/PD-1-mediated T-cell exhaustion, FABP5-mediated lipid uptake, FA), MDSC recruitment via the CXCL2/CXCR2 axis, exosome-mediated immune regulation, and NK cell regulation.

Schematic overview of CLEVER-1^+^ TAMs in the gastric cancer microenvironment. Immunosuppressive signals drive CLEVER-1 upregulation and polarization of macrophages toward an M2-like phenotype. These cells undergo metabolic reprogramming characterized by enhanced FAO via FABP5, high ARG1 expression, and SLC2A1 (GLUT1)-mediated glycolysis (preliminary evidence in gastric cancer). The resulting immunosuppressive outputs include direct CD8^+^ T-cell inhibition through PD-L1/PD-1 engagement and ARG1 activity, secretion of IL-10, TGF-β, and VEGF, recruitment of myeloid-derived suppressor cells via the CXCL2/CXCR2 axis (hypothesized), and induction of CD8^+^ T-cell exhaustion. Potential therapeutic intervention points comprise anti-CLEVER-1 antibody (bexmarilimab), ARG1 inhibitor (e.g., CB-1158), FAO inhibitor (etomoxir, requiring further validation), and combinatorial strategies such as anti-PD-1, TGF-β inhibitors, or CD47-SIRPα blockade.
